# Human Infants and Baboons Show the Same Pattern of Handedness for a Communicative Gesture

**DOI:** 10.1371/journal.pone.0033959

**Published:** 2012-03-21

**Authors:** Helene Meunier, Jacques Vauclair, Jacqueline Fagard

**Affiliations:** 1 Primatology Centre of Strasbourg University, Fort Foch, Niederhausbergen, France; 2 Research Center in the Psychology of Cognition, Language and Emotion, Aix-Marseille University , Aix-en-Provence, France; 3 Laboratoire Psychologie de la Perception, Université Paris Descartes, Paris, France; Katholieke Universiteit Leuven, Belgium

## Abstract

To test the role of gestures in the origin of language, we studied hand preferences for grasping or pointing to objects at several spatial positions in human infants and adult baboons. If the roots of language are indeed in gestural communication, we expect that human infants and baboons will present a comparable difference in their pattern of laterality according to task: both should be more right-hand/left-hemisphere specialized when communicating by pointing than when simply grasping objects. Our study is the first to test both human infants and baboons on the same communicative task. Our results show remarkable convergence in the distribution of the two species' hand biases on the two kinds of tasks: In both human infants and baboons, right-hand preference was significantly stronger for the communicative task than for grasping objects. Our findings support the hypothesis that left-lateralized language may be derived from a gestural communication system that was present in the common ancestor of baboons and humans.

## Introduction

One of the keys to understanding language evolution is the issue of hemispheric specialization in the communicative behaviors of non-human primates. While left lateralization for language is classically linked to right-handedness for manipulative actions in humans, a different approach [1] suggests that handedness for gestural communication may constitute a better predictor of hemispheric specialization for language. This view is supported by the existence of strong links between speech and gestures in humans [2]. The aim of our study is to look for possible convergence between non-human primates and infants with respect to the laterality of gestural communication.

The facts (1) that nonhuman primates' gestures (*e.g.*, pointing in the chimpanzee) also convey intentional and relational content [3], and (2) that great apes [4], monkeys [5], as well as (3) human infants [6], [7] show preferential use of the right hand when pointing, indicate that non-human primates and humans who have not yet acquired language are ideal models to investigate language precursors. However, comparative data on this question is critically lacking. Our study aims to overcome this deficit by testing human infants and nonhuman primates using a similar experimental setup. For this purpose we adapted a test initially designed to quantify hand preference in humans [8], so it could be used to investigate both human infants and adult baboons. This experimental paradigm allowed us to assess handedness in both species in a communication task *versus* a simple (manipulative) grasping task, while controlling spatial and postural factors.

If the roots of language are indeed in gestural communication, we expected that human infants and baboons would present a comparable difference in the pattern of laterality according to the task: both should be more right-hand/left-hemisphere specialized when communicating by pointing than when simply grasping objects. We also predicted that the position of laterally presented objects would influence hand choice for pointing to laterally presented items, but to a lesser extent than for object grasping.

## Methods

This experiment included only behavioral observations, routine training and non-invasive contact with both the infants and the baboons. The infant experiment was conducted in accordance with the ethical standards specified in the 1964 declaration of Helsinki, and written formal parental consent was granted before observation. Our institutional review boards approved this study for both infants and baboons (authorization number for experimentation on baboons: C 13-087.7).

### Subjects

The subjects were 12 captive Olive baboons (*Papio anubis*) including 2 adult females (8 and 17 years old), 9 adult males (6, 6, 7, 8, 9, 9, 11, 12 and 12 years old) and 1 subadult male (4 years old). All the subjects lived in social groups, and were housed either in parks or large cages, both with free access to an indoor shelter. Individuals participated spontaneously in the experiments, so our subjects are mainly dominant individuals from each group.

We also tested 10 infants on three occasions, i.e., at 14, 17 and 20 months of age. This age range was chosen because it has been shown that infants start to point around the end of the first year [9] and that about two thirds of infants point at 14 months [10]. We stopped at 20 months since we did not want to test pointing gestures accompanying language and since many infants undergo a language spurt at the end of the second year [11]. For the infants, we have no missing data on either task. Thirteen infants were initially part of the study. For one of them, there was one trial missing for pointing, for another, there were 2 trials missing for pointing, and for the third one, there were 5 trials missing for the grasping task. There was no difference in the significance of the results with and without these three infants. We therefore decided to be conservative and keep only the infants for whom there was no missing data.

### Training

For the baboons, a training phase was necessary until each subject was able to point at one baited container with its whole hand. Initially, a reachable raisin was presented to the subject on the experimental table so that the subject was able to grasp it. Next, the raisin was progressively moved further away up to an ambiguous distance (raisin was almost reachable) so that when the subject attempted to grasp the raisin, the experimenter gave it directly to the animal. Finally, the raisin was placed at 70 cm from the subject, i.e., beyond the subject's reach. Training was terminated when the subject was able to point at the out-of-reach raisin with one hand (left or right) and without trying to grasp it.

### Previous experience of the subjects

Four of our adult males baboons had already participated in experiments in which they were trained to point toward unreachable food using the same methodology (see [5]) as the one described above.

### Experimental procedure

**Figure 1:**
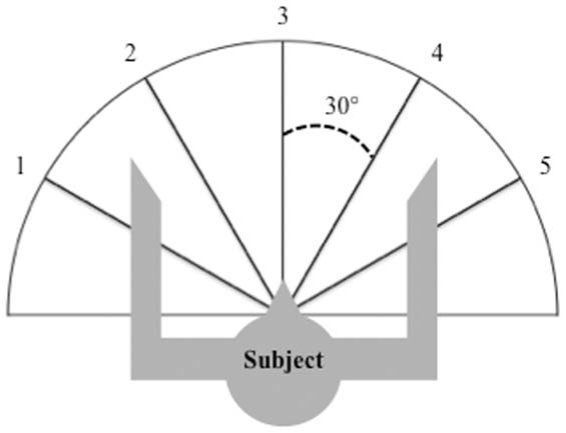
Experimental setup for grasping.

For the grasping task, an attractive item (a toy for infants and a raisin for baboons) was placed in a randomized order at one of five positions, each separated from the adjacent position(s) by 30° on a half-circle, at a distance reachable by each subject's hand (Figure 1). For the pointing task with infants, we presented puppets through holes made in a white sheet lining the wall facing the infant, with an angle of 20° between adjacent holes. Infants were seated at a distance of two meters from the screen, between a parent and an observer who encouraged them to indicate the puppet when they did not point spontaneously. For pointing in baboons, an opaque container in which raisins were hidden was placed at each of the five positions. Only one of the five containers was baited at a time. Two experimenters performed the task. The first experimenter hid raisins in one of the five containers. The second experimenter was present, but could not see where the raisins were hidden. Then the second experimenter faced the baboon, who then had to point toward the correct container so the experimenter would retrieve the raisin for the baboon. Two experimenters were used for the baboons in order to avoid any ambiguity concerning the production and the interpretation of the gesture performed by the monkey, given that Experimenter 2 had no knowledge of the baited position and had to rely solely on the cues provided by the animal. Above all, the use of 2 experimenters allowed us to impose a delay in the production of the pointing gesture, and consequently it facilitated discrimination between a response that could be considered as a “frustrated reaching response” from an intentional communicative gesture. If the subject pointed to the correct container, it was rewarded with the food placed under the container. In the rare cases when the subject pointed to another container, the food was removed, the trial was cancelled and a new trial started. We noted the hand used for grasping and pointing in both infants and baboons. We recorded 5 trials per position and per subject for grasping and 3 trials per position and per subject for pointing. We then compared hand-biases for each species and task.

## Results

**Figure 2:**
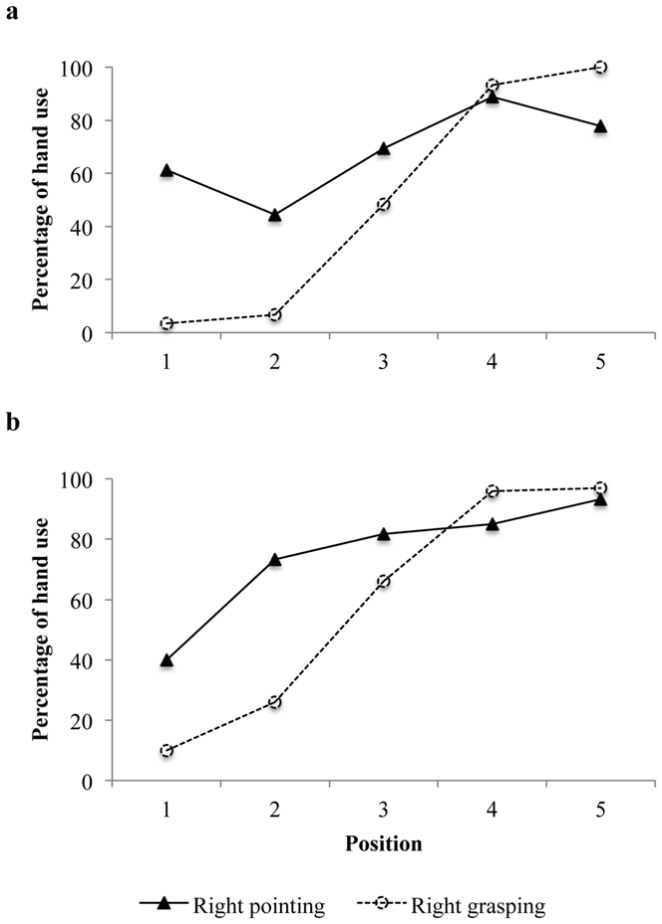
Percentage of right hand use for each task in baboons (a) and human infants (b).

An ANOVA calculated on the handedness index HI = (Number of Right Hand - Left Hand choices)/(Number of Right Hand + Left Hand choices) as a function of group (human infants versus baboons), with position (×5) and task (×2) as repeated measures, showed a main effect of position (F(4,80) = 61.86, *P*<0.0000001), a main effect of task (F(1,20) = 13.9, *P*<0.01), a task x position interaction (F(4,80) = 15.38, *P*<0.000001), but no task x group interaction and no task x position x group interaction (Figure 2). There was also no group effect (*P* = 0.29). A post-hoc Tukey test showed that the two tasks differed significantly for the two left positions (p<.001 for both). We also checked for an age effect in infants. An ANOVA on HI at the three sessions (repeated measures, 14, 17 and 20 months) showed no main effect of age (F(2,18) = 1.5, p = .25) and no age x task interaction (F(2,18) = .77, p = .48).

## Discussion

Overall, the results show a remarkable convergence in the the distribution of the two species' hand biases on the two kinds of tasks. When grasping, both infants' and baboons' hand preferences were shown to depend on item position as previously reported [7], [12]. Subjects reached for spatial positions located to the right of their body's midline (positions 4 and 5) predominantly with their right hand, and positions situated to the left (positions 1 and 2) predominantly with their left hand. By contrast, hand preferences for the pointing task significantly favored the use of the right hand in the two species. The greater right hand preference for communicative gestures compared to object manipulation concurs with previous reports concerning pointing gestures, symbolic gestures, and ASL signs in infants and toddlers [6], [13], [14] but also with reports concerning gestural communication in nonhuman primates [15], [16]. Moreover, according to Taglialatela, Cantalupo, and Hopkins [17], right-handedness for food-begging gestures in chimpanzees is associated with morphological left asymmetries in the homologue of Broca's area (inferior frontal gyrus).

Taken together, these findings support the hypothesis that left lateralization for language may be derived from a gestural communication system that was present in the common ancestor of baboons, chimpanzees and humans. Vocalizations, although late-comers in the evolution of intentional communication, may have gradually invaded this gestural system in the course of evolution [18], eventually leading to the dominance of the vocal modality (speech) in humans.
